# Fatty acid extract from CLA-enriched egg yolks can mediate transcriptome reprogramming of MCF-7 cancer cells to prevent their growth and proliferation

**DOI:** 10.1186/s12263-016-0537-z

**Published:** 2016-07-27

**Authors:** Aneta A. Koronowicz, Paula Banks, Dominik Domagała, Adam Master, Teresa Leszczyńska, Ewelina Piasna, Mariola Marynowska, Piotr Laidler

**Affiliations:** 1 Department of Human Nutrition, Faculty of Food Technology, University of Agriculture, Krakow, Poland; 2 Department of Biochemistry and Molecular Biology, Medical Centre for Postgraduate Education, Warsaw, Poland; 3 Department of Medical Biochemistry, Jagiellonian University Medical College, Krakow, Poland

**Keywords:** AKT/mTOR pathway, CLA-enriched egg yolks, DNA microarray, Cancer chemoprevention, MCF-7 cancer cells, Transcriptome, SFA/MUFA

## Abstract

**Background:**

Our previous study showed that fatty acids extract obtained from CLA-enriched egg yolks (EFA-CLA) suppressed the viability of MCF-7 cancer cell line more effectively than extract from non-enriched egg yolks (EFA). In this study, we analysed the effect of EFA-CLA and EFA on transcriptome profile of MCF-7 cells by applying the whole Human Genome Microarray technology.

**Results:**

We found that EFA-CLA and EFA treated cells differentially regulated genes involved in cancer development and progression. EFA-CLA, compared to EFA, positively increased the mRNA expression of *TSC2* and *PTEN* tumor suppressors as well as decreased the expression of *NOTCH1*, *AGPS*, *GNA12*, *STAT3*, *UCP2*, *HIGD2A*, *HIF1A*, *PPKAR1A* oncogenes.

**Conclusions:**

We show for the first time that EFA-CLA can regulate genes engaged in AKT/mTOR pathway and inhibiting cell cycle progression. The observed results are most likely achieved by the combined effect of both: incorporated CLA isomers and other fatty acids in eggs organically modified through hens’ diet. Our results suggest that CLA-enriched eggs could be easily available food products with a potential of a cancer chemopreventive agent.

**Electronic supplementary material:**

The online version of this article (doi:10.1186/s12263-016-0537-z) contains supplementary material, which is available to authorized users.

## Introduction

Conjugated linoleic acid (CLA) term includes several isomers of linoleic acid (18:2), naturally present in ruminant and dairy products, due to the activity of the rumen microflora [25, 30]. In numerous studies, CLA was shown to have several beneficial properties on human health. Researchers examined its effect on stimulating the immune system [1], reducing cancerogenesis [32, 52], atherogenesis [34], diabetes, and obesity [54]. However, most of the available literature was focused on the activity of isolated, pure substances. In addition, according to available data, the consumable quantities of naturally occurring CLA are relatively too low to effectively impact human health [27]. The recommended effective dose of CLA was estimated at least 1.5–3.5 g/day [13], while natural ruminant products contain between 1.2 and 12.5 mg per gram of fat [25, 30, 47, 78] and in poultry CLA concentration remains relatively low, at 0.6 to 0.9 mg per gram of fat [13]. Enhancing CLA concentration in food products such as eggs, chosen dairy products/yogurts, and meat could then become an alternative to synthetic CLA supplements. Indeed, studies have shown an easy incorporation of CLA into eggs of chickens by diet fortification [11, 65, 74] and that CLA-enriched eggs meet the requirements of functional food [24].

Little is known about the effect of fatty acids (FA) from CLA-enriched food products on cancer cells [16, 46]. Our previous study showed that FA extracts obtained from CLA-enriched egg yolks (EFA-CLA) suppressed the viability of MCF-7 cancer cell line more effectively than the extracts from non-enriched egg yolks (EFA) [32]. To analyze the potential molecular mechanism, we decided to compare the effects of both extracts on MCF-7 cells transcriptome profile.

The whole-genome DNA microarray technology has become a very powerful tool to analyze global gene expression profiles, and in multiple studies, it has been shown to be an effective method for detecting genomic variation of closely related samples. Finally, we identified and analyzed differently expressed genes based on family, molecular functions, biological processes, cellular components, or pathways. As suggested in this article, relationships between studied genes require a confirmation at protein level; nevertheless, the microarray results are a valuable and multi-faceted source of information for other scientists and a foundation for further in vivo research [67].

## Methods

### Hens’ and eggs’ management

The Animal Ethics Committee of the National Institute of Animal Production (Poland) approved all experiments involving animals (approval number: 851/2011). All applicable international, national, and/or institutional guidelines for the care and use of animals were followed.

Forty-eight *Isa Brown* laying hens (26 weeks old) were housed in a controlled room under 14/10 h light/dark cycle, given free access to water and commercial starter diet (‘DJ’ feed). After a 1-week adaptation period, an equal number of hens was randomly allocated to the control or experimental group for 4 months of the experiment. Diets (Additional file 1: Table S1) were calculated to provide 2700 kcal/kg and 17 % crude protein. The 0.75 % dietary CLA (TONALIN FFA 80, BASF Company, Germany) concentration was based on previously determined formula [24] and contained 80 % CLA in 50:50 ratio for *cis-9*,*trans-11* and *trans-10*,*cis-12* isomers. Eggs were collected daily for the period of 10 weeks and stored at 4 °C. Yolks were separated from albumen, homogenized with rotary homogenizer, and frozen at −20 °C. Samples were then lyophilized (Martin Christ Model Alpha 1–4, Germany) and again stored at −20 °C. The total dry matter was determined by oven drying method [3] and the total fat content was determined by Soxhlet method (Soxtec Avanti’s 2050 Auto Extraction Unit, Tecator Foss, Sweden) using petroleum ether as a solvent [23].

### Fatty acids extraction and GC/MS analysis

Lipids from control and CLA-enriched yolks were extracted by using modified Folch method [22]. One gram per liter of butylated hydroxytoluen (BHT) was used as an antioxidant. Briefly, after overnight incubation with chloroform/methanol (2:1) solution, samples were filtrated and mixed with 4 mL of 0.88 % sodium chloride solution to obtain phase separation. Chloroform lipids layer was then carefully dried under nitrogen. Ten milligrams of each lipid extract was subjected to saponification (20 min, 60 °C) with 0.5 M KOH/methanol followed by methylation with 14 % (*v*/*v*) BF3/methanol (15 min, 60 °C) and extraction with hexane. The obtained fatty acid methyl esters (FAME) were analyzed by GC/MS (Additional file 2: Table S2). The profile of EFA-CLA and EFA was expressed as percentage (%) of relative area, obtained by area normalization (FA peak area relative to chromatogram total area). For the treatment, lipid extracts were subjected to the basic hydrolysis (0.5 M KOH, 60 °C, 15 min) and extracted with hexane. The free fatty acids were then dissolved in ethanol at the stock concentration 1 g/mL and stored under nitrogen in the temperature of −20 °C.

### Cell culture and treatment

Human breast adenocarcinoma cell line MCF-7 (ATCC^®^ HTB­22^TM^) was purchased from the American Type Culture Collection. Cells were cultured according to the manufacturer’s procedure. Cells were seeded in culture plates (BD Biosciences) for 24 h. After that time, growing medium was replaced by a medium containing (a) fatty acid extract from CLA-enriched egg yolks (EFA-CLA) and (b) fatty acids extract from non-enriched egg yolks (EFA), both at the concentration of 0.5 mg/mL. We used (c) cell cultures only in growth medium (empty control (EC)) and (d) cell cultures treated with only a solvent of fatty acids (ET-ethanol) at final concentration 0.1 %, as a negative control (NC).

### Cell proliferation

Cell proliferation was determined with 5′-bromo-2′-deoxy-uridine (BrdU) Labeling and Detection Kit III (Roche), according to manufacturer’s instruction.

### Microarray analysis of gene expression profile

Whole Human Genome Microarrays containing about 50 000 probes (Agilent Technologies, USA) were used to establish the expression profile of each tested sample. Total RNA was isolated from cells using RNA isolation kit (A&A Biotechnology, Poland). RNA quantity was measured with NanoDrop (NanoDrop Technologies, USA). The analysis of its quality and integrity was performed with BioAnalyzer (Agilent, USA). Only samples with RNA integrity number (RIN) ≥8.0 were included in the analysis which was performed using SurePrint G3 Human Gene Expression 8x60K v2 Microarray. Each slide contained eight microarrays representing about 50 000 probe sets. The Low Input Quick Amp Labeling Kit, two-color (Agilent, USA) was used to amplify and label target RNA to generate complementary RNA (cRNA) for oligo microarrays used in gene expression profiling. The experiment was performed using a common reference design, where the common reference was a pool of equal amounts of RNA from control cells. On each of two-color microarrays, 300 ng of cRNA from the pool (labeled Cy3) and 300 ng of cRNA (labeled Cy5) were hybridized. In total, 12 microarrays were run—three for each experimental group. Microarray hybridization was performed with the Gene Expression Hybridization Kit (Agilent Technologies, USA), according to the manufacturer’s protocols. RNA Spike In Kit (Agilent Technologies, USA) was used as an internal control. Acquisition and analysis of hybridization intensities were performed using the Agilent DNA microarray scanner. Data were extracted and background was subtracted using standard procedures contained in the Agilent Feature Extraction (FE) Software version 10.7.3.1. FE performs Lowess normalization. Samples underwent quality control and the results showed that each sample had a similar QC metric profile. The next step was filtering probe sets by flags to remove poor quality probes (absent flags). Microarray data were deposited at the Gene Expression Omnibus data repository under the number GSE65397 and followed MIAME requirements. To identify signaling pathways and gene functions, the microarray data were analyzed using Panther Classification System—an online database.

### RT and real-time PCR analysis

Reverse transcription was performed using 1 μg of total RNA isolated from the cells by using the Maxima first Strand cDNA Synthesis kit for RT-qPCR (Thermo Scientific). A quantitative verification of genes was performed using the CFX96 Touch™ Real-Time PCR Detection System instrument (Bio-Rad), utilizing the SYBR Green Precision Melt Supermix kit (Bio-Rad). Conditions of individual PCR reactions were optimized for given pair of oligonucleotide primers (Additional file 3: Table S3). Basic conditions were as follows: 95 °C for 10 min, 45 PCR cycles at 95 °C, 15 s; 59 °C, 15 s; 72 °C, 15 s, followed by melting curve analysis (65–97 °C with 0.11 °C ramp rate and five acquisitions per 1 °C). Results were normalized using at least two reference genes (*GAPDH*, *HPRT1*, *ACTB*, or *HSP90AB1*) and were calculated using the 2^−∆∆C^T method [39].

### Statistical analysis

Each treatment included three replicates and the experiment was repeated three times. Statistical analysis for microarrays was performed using Gene Spring 12.6.1 software (Agilent, USA). Statistical significance of the differences was evaluated using a one-way ANOVA and Tukey’s HSD Post hoc test (*p* < 0.05). A multiple testing correction was performed using Benjamini and Hochberg false discovery rate (FDR) <5 %. Other experiments were assessed by Student’s *t* tests.

## Results

### Bird performance and egg composition

**Fig. 1:**
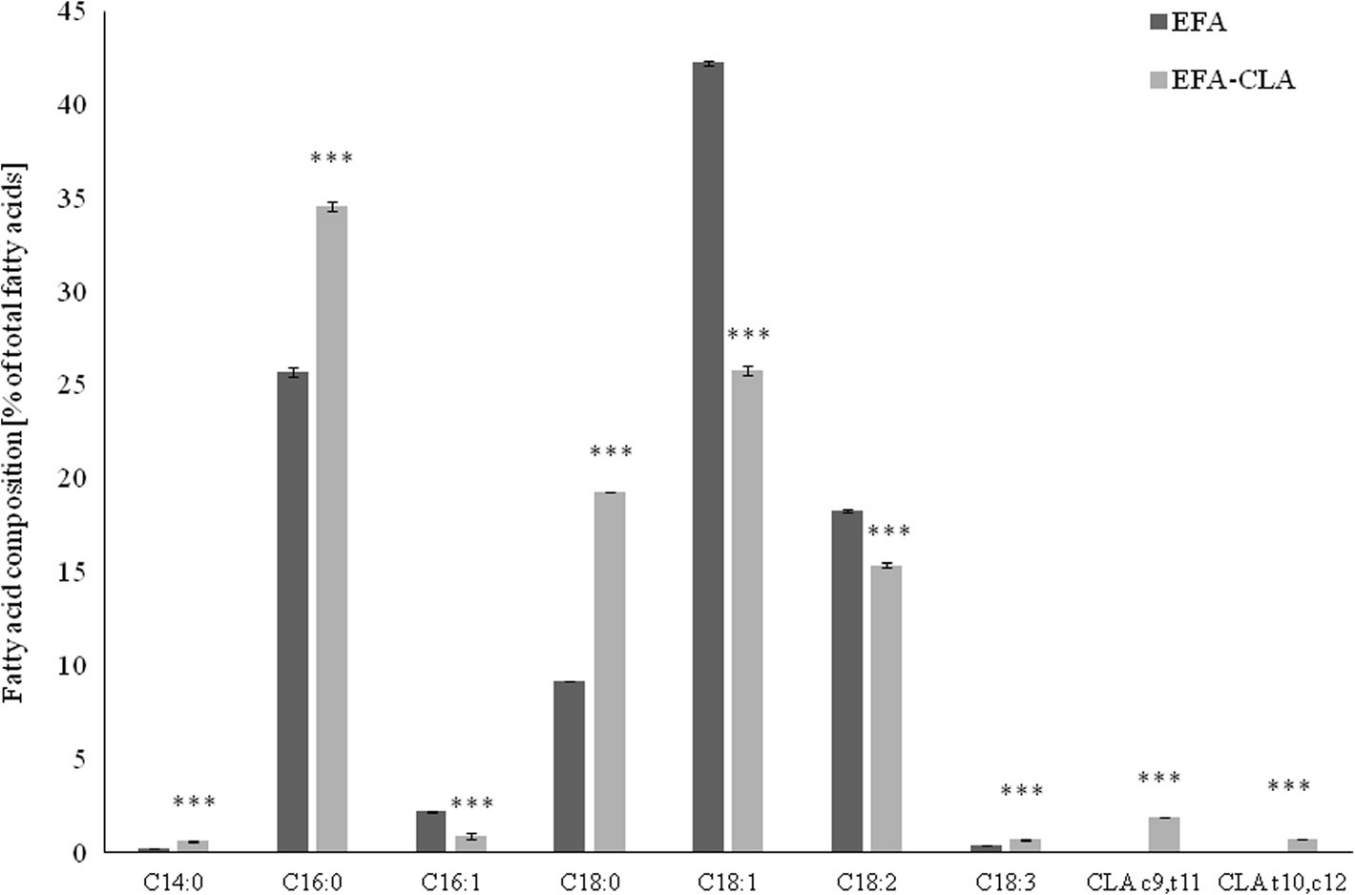
Effect of dietary CLA on relative (%) fatty acids composition of egg yolks. Statistical significance of treatment: *p < 0.05; **p < 0.01; ***p < 0.001

There were no significant differences in egg production between hens as well as egg characteristics [33]. Fatty acid profile in CLA-enriched egg yolks was significantly affected by dietary CLA fortification (Fig. 1). Both CLA isomers were found incorporated into the yolk, and their concentration did not reflect their initial proportion in the experimental diets (Additional file 1: Table S1), with the preference for the *cis-9*,*trans-11* isomer. Compared to the control, feeding with 0.75 % of dietary CLA significantly increased (*p* < 0.001) total SFA concentration at the expenses of MUFA (*p* < 0.001) (Fig. 1 and [33]).

### Cell proliferation

**Fig. 2:**
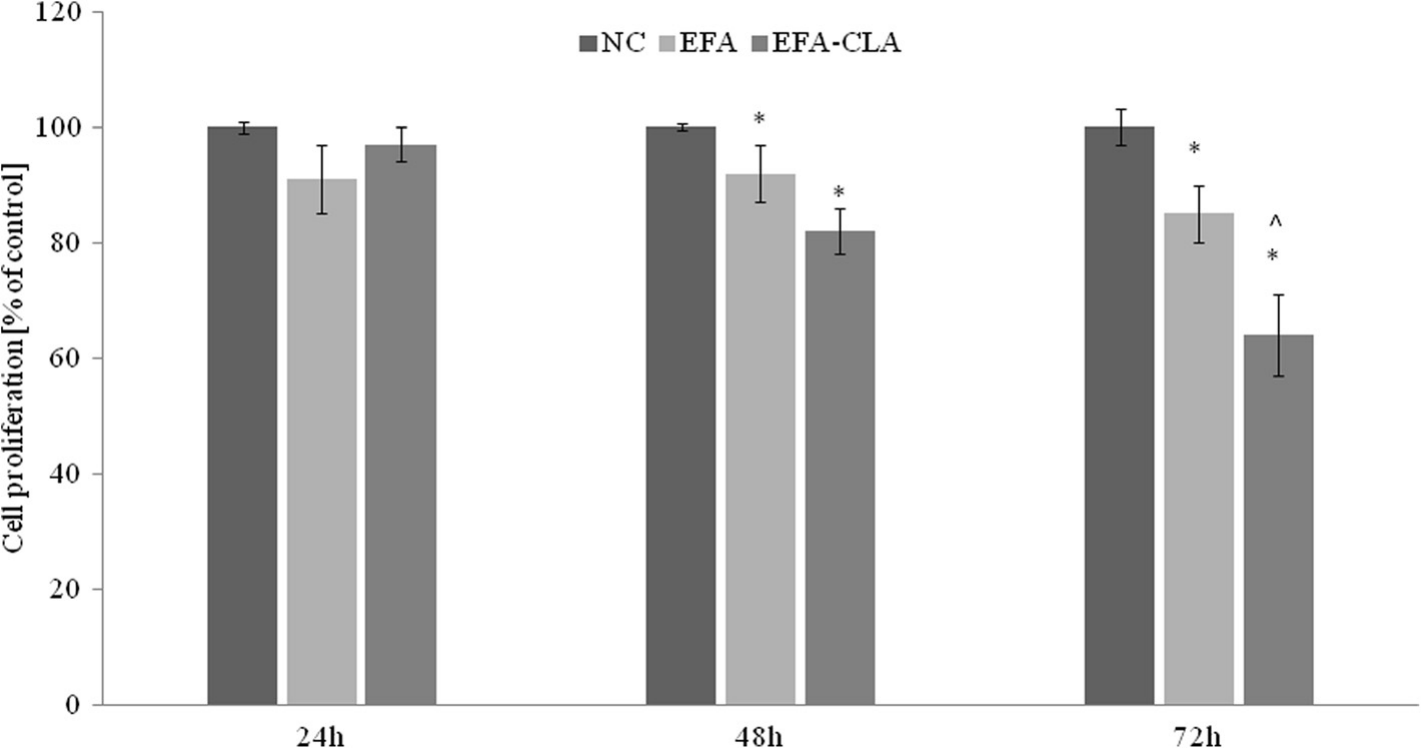
Effect of EFA-CLA on MCF-7 cells proliferation. Values are expressed as means ± SEM for the *N* ≥ 9, standarized to NC as 100 %. Statistical significance was based on Student’s *t* test *p < 0.05 vs. NC and ^*p* < 0.05 vs. EFA

**Fig. 3:**
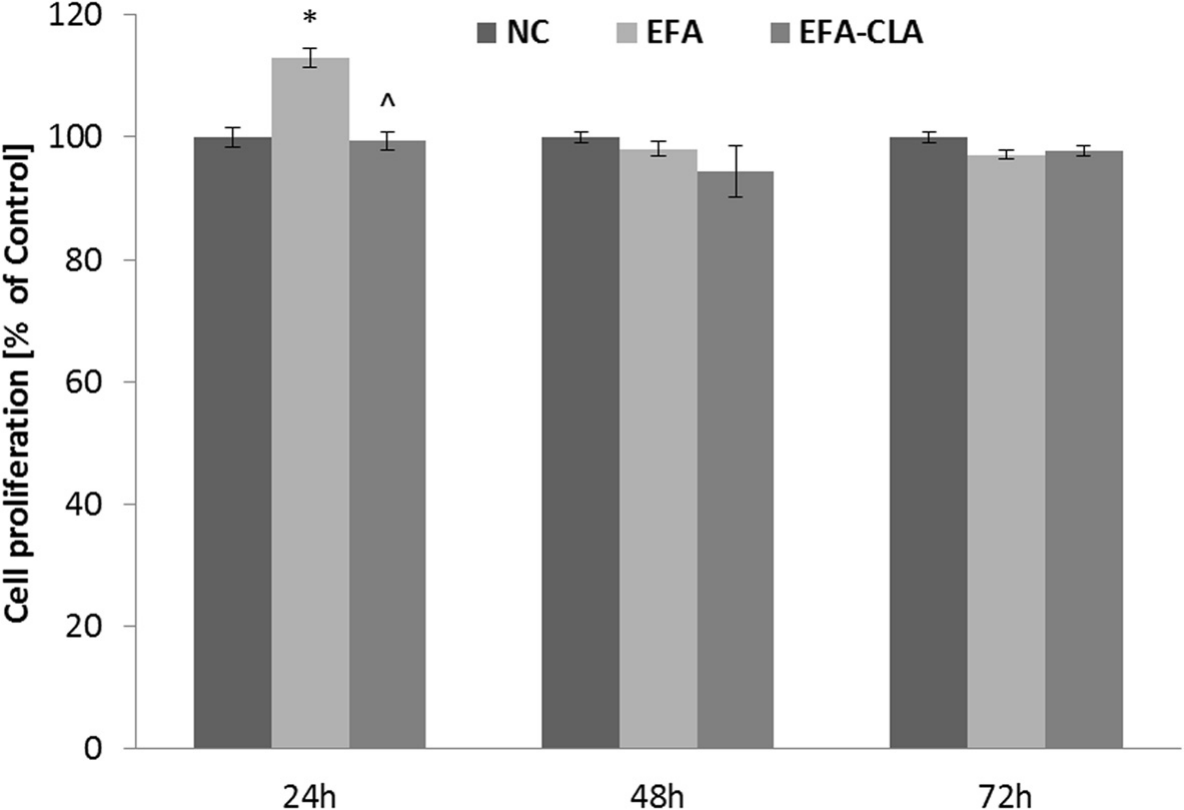
Effect of EFA-CLA on MCF-10A cells proliferation. Values are expressed as means ± SEM for the *N* ≥ 9, standarized to NC as 100 %. Statistical significance was based on Student’s *t* test *p < 0.05 vs. NC and ^*p* < 0.05 vs. EFA

EFA-CLA extract at a concentration of 0.5 mg/mL suppressed MCF-7 cell proliferation more effectively than the extract from non-enriched egg yolks. Specifically, treatment with EFA-CLA reduced the cell proliferation by approximately 40 % compared to the negative control, while EFA reached 20 % (Fig. 2). Moreover, this effect was weaker for estrogen-negative MDA-MB-231 (Additional file 4: Figure S12) and not observed for a non-tumorigenic MCF-10A cell line (Fig. 3).

### Effect of applied treatments on MCF-7 cell line transcriptome profile

**Fig. 4:**
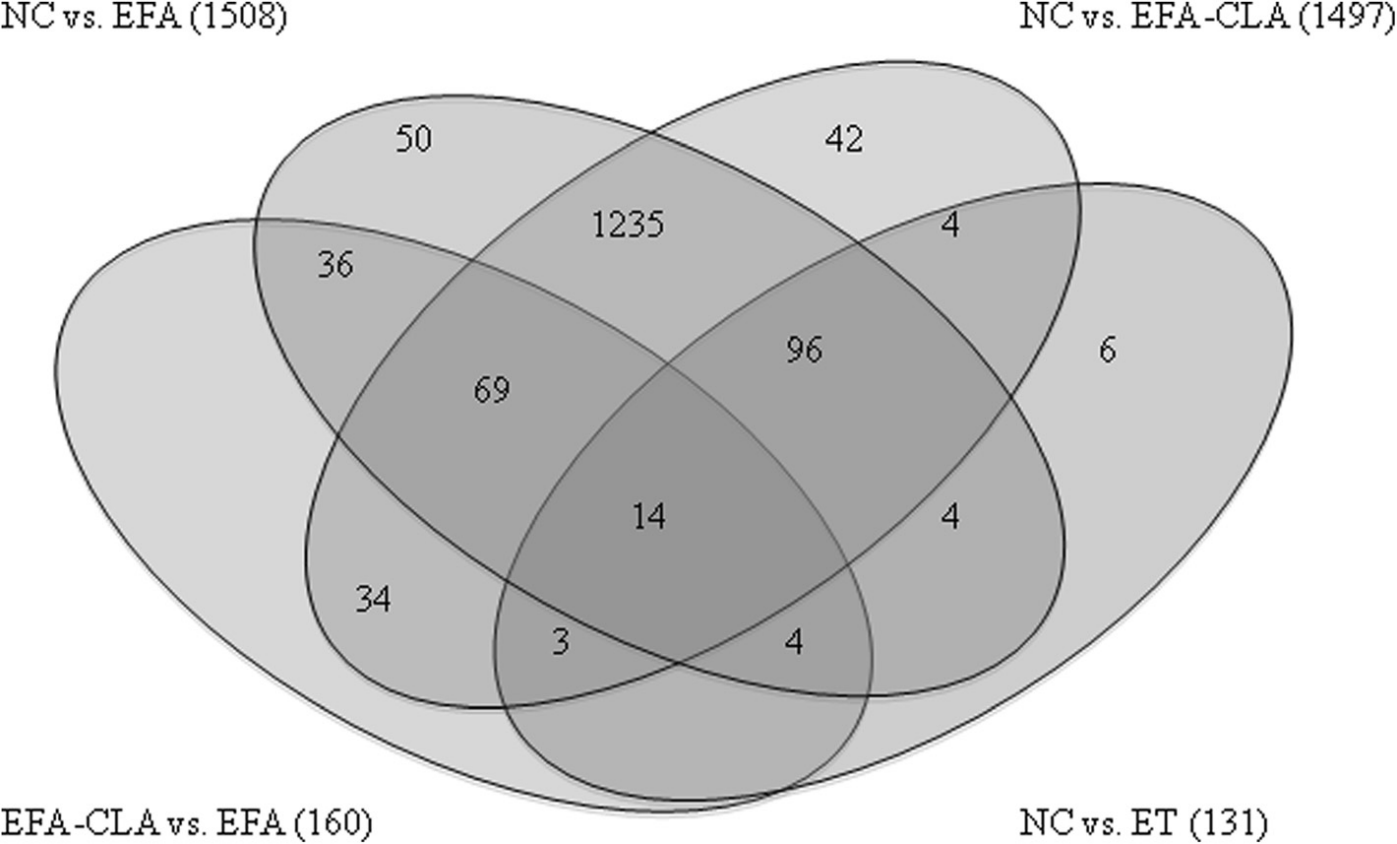
Analysis of differently expressed transcripts between experimental groups

The analysis was performed on 1589 transcripts, of which 160 were differently expressed between EFA-CLA and EFA studied groups (Fig. 4 and Additional file 5: Table S4). We omitted 21 genes, which could have been directly affected by the solvent (ET) leaving 139 genes (Additional file 6: Table S10). Further analysis showed 69 transcripts shared by all three comparison groups: EFA-CLA vs. EFA, NC vs. EFA-CLA, and NC vs. EFA and 36 transcripts shared by EFA-CLA vs. EFA and NC vs. EFA. We determined 34 transcripts unique only to EFA-CLA vs. EFA. Among those, 11 were uncharacterized in available databases. Finally, we identified 18 (underlined) that, according to the available data, can be linked to cancer development and/or progression and which are involved in important cellular processes including regulation of cell cycle, apoptosis, or cell metabolism (Table 1). For those, the differences in expression between EFA and NC were statistically insignificant (Additional file 7: Table S5).

**Table 1:** The list of the differently regulated EFA-CLA vs. EFA specific transcripts in MCF-7 cell line

Gene Symbol	Adjusted *p* values	FC value	Gene name
***NOTCH1***	0.0146	−2.63	Notch homolog 1, translocation-associated
***AGPS***	0.0424	−2.19	Alkylglycerone phosphate synthase
***GNA12***	0.0009	−1.56	Guanine nucleotide binding protein (G protein) alpha 12
***HIF1A***	0.0184	−1.56	Hypoxia inducible factor 1, alpha subunit (basic helix-loop-helix transcription factor)
***STAT3***	0.0088	−1.32	Signal transducer and activator of transcription 3 (acute-phase response factor)
***UCP2***	0.0083	−1.29	Uncoupling protein 2 (mitochondrial, proton carrier)
***HIGD2A***	0.0089	−1.27	HIG1 hypoxia inducible domain family, member 2A
*WASH1*	0.0038	−1.27	WAS protein family homolog 1
***BIN3***	0.0477	−1.16	Bridging integrator 3
***PRKAR1A***	0.0462	−1.14	Protein kinase, cAMP-dependent, regulatory, type I, alpha
*NDUFB11*	0.0105	−1.13	NADH dehydrogenase (ubiquinone) 1 beta subcomplex, 11, 17.3 kDa
***ANXA5***	0.0311	−1.05	Annexin A5
***SMS***	0.0399	1.08	Spermine synthase
***PPP2R5E***	0.0009	1.13	Protein phosphatase 2, regulatory subunit B', epsilon isoform
***NAP1L1***	0.0335	1.14	Nucleosome assembly protein 1-like 1
***PTEN***	0.0382	1.15	Phosphatidylinositol 3,4,5-trisphosphate 3-phosphatase and dual-specificity protein phosphatase PTEN
*LOC646214*	0.0213	1.16	p21 protein (Cdc42/Rac)-activated kinase 2 pseudogene
***LMCD1***	0.0067	1.21	LIM and cysteine-rich domains 1
***CAMSAP2***	0.0174	1.23	Calmodulin regulated spectrin-associated protein family, member 2
***TSC2***	0.0177	1.26	Tuberous sclerosis 2
*FLJ45139*	0.0406	1.33	FLJ45139 protein
*CHSY3*	0.0399	1.72	Chondroitin sulfate synthase 3
***OVOS***	0.0403	1.72	Ovostatin
***SCD***	0.19	−1.18^NS^	Stearoyl-CoA desaturase (delta-9-desaturase)

Statistical significance of treatment: *p* < 0.05; NS *p* > 0.05; bolded genes are to be associated with cancer

### Real-time PCR

To validate microarray data, we selected eight random genes from EFA-CLA vs. EFA comparison group: *NOTCH1*, *HIGD2A*, *PPKAR1A*, *UPC2*, *NAP1L1*, *CAMSAP2*, *PPP2R5E*, and *TSC2* (*p* < 0.05, Table 2). Our analysis showed a significant decrease in the messenger RNA (mRNA) expression of *NOTCH1*, *HIGD2A*, *PPKAR1A*, and *UCP2* and an increase in the expression of *CAMSAP2*, *PPP2R5E*, and *TSC2* genes due to the EFA-CLA treatment. Interestingly, only RT-qPCR results for *NAP1L1* exhibited changes in the opposite direction than the microarray.

**Table 2:** The validation of microarray results with RT-qPCR in MCF-7 cell line (EFA-CLA vs. EFA)

Gene symbol	Adjusted *p* values	FC value RT-qPCR	FC value Microarray	Gene name
*NOTCH1*	0.0022	−2.02	−2.63	Notch homolog 1, translocation-associated
*HIGD2A*	0.0121	−1.75	−1.27	HIG1 hypoxia inducible domain family, member 2A
*PRKAR1A*	0.0047	−1.58	−1.14	Protein kinase, cAMP-dependent, regulatory, type I, alpha
*UCP2*	0.0023	−1.53	−1.29	Uncoupling protein 2 (mitochondrial, proton carrier)
*NAP1L1*	0.0023	−1.37	1.17	Nucleosome assembly protein 1-like 1
*CAMSAP2*	0.0031	1.57	1.23	Calmodulin regulated spectrin-associated protein family, member 2
*PPP2R5E*	0.0035	1.72	1.13	Protein phosphatase 2, regulatory subunit B', epsilon isoform
*TSC2*	0.0001	1.85	1.26	Tuberous sclerosis 2

Statistical significance of treatment: *p* < 0.05

### GO molecular complete analysis

Next, we examined the Gene Ontology (GO) for EFA-CLA vs. EFA differently regulated genes, using Panther Classification System. Results obtained from analysis of the signaling pathways, biological processes, molecular functions, and protein classes are presented in supplementary material (Additional file 8: Table S6, Additional file 9: Table S7, Additional file 10: Table S8, and Additional file 11: Table S9).

### Effect of EFA-CLA on oncogenic pathways

**Fig. 5:**
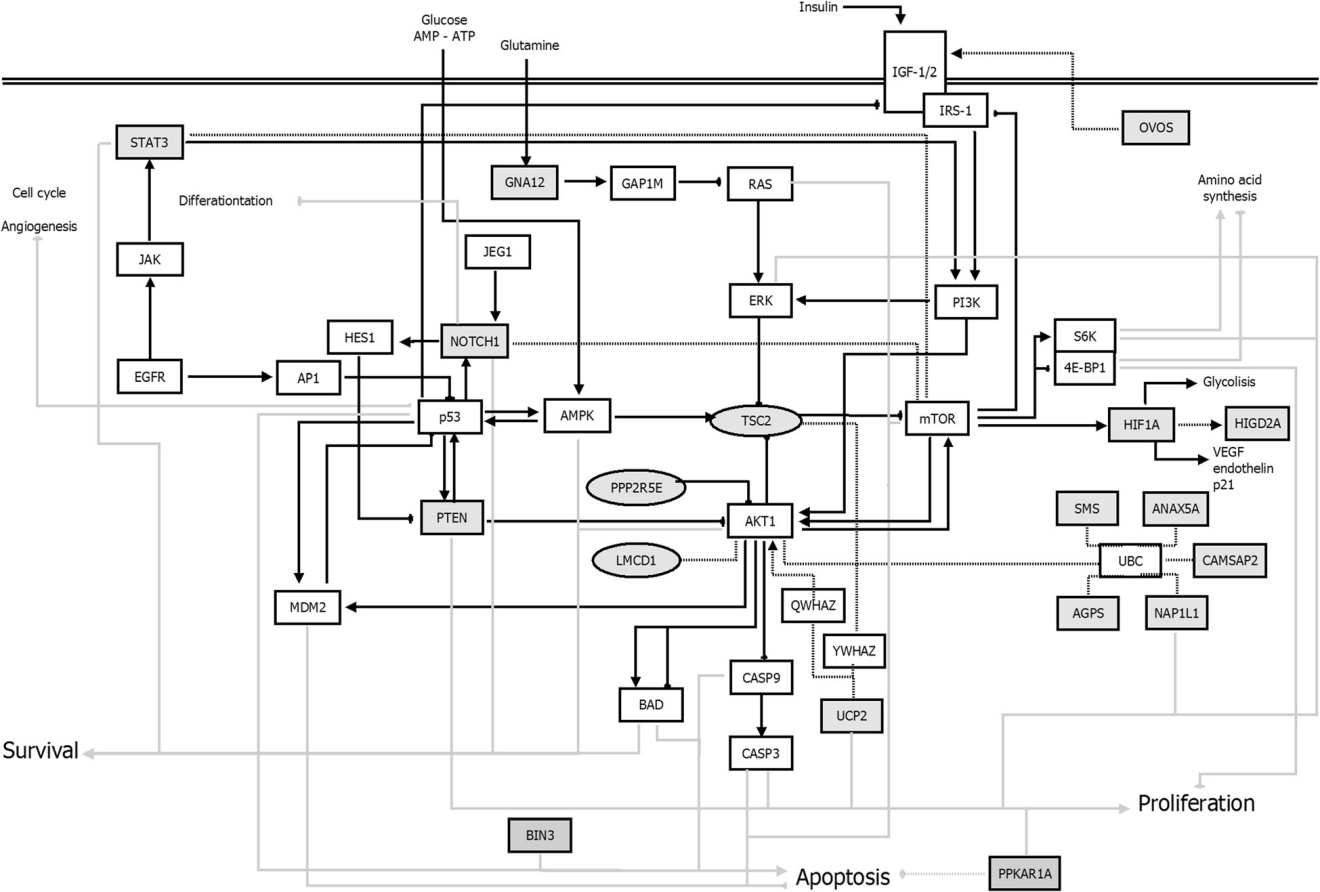
Potential interactions between differently regulated genes treated with EFA-CLA in MCF-7 cells (listed in Table 1)

In addition to signaling pathways listed in Additional file 8: Table S6, we aimed to study the connections between all the transcripts from Table 1, especially in terms of oncogenic pathways (Fig. 5) [79]. Our results showed that EFA-CLA treatment affected the downstream genes of the mammalian target of rapamycin (mTOR) signaling pathway. The increased mRNA expression of *PTEN*, *PPP2R5E*, and *TSC2* and decreased expression of *GNA12*, *UPC2*, *AGPS*, *ANAX5A*, and *HIF1A*, together with observed reduced proliferation of MCF-7, suggest that EFA-CLA negatively regulates AKT/mTOR pathway.

## Discussion

Breast cancer is one of the most common malignancies among women [21]; however, despite extensive research, the cellular processes that lead to carcinogenesis have not yet been fully explained. In the current research, we chose the estrogen receptor-positive (ER+) MCF-7 breast cancer cell line—the most studied cellular model of breast cancer [26, 59, 73]. One of the main reasons behind our choice is that the (ER+) breast cancers are the most frequently diagnosed breast cancer subtype.

CLA is an extensively studied compound, and research findings showed a variety of possible beneficial effects of dietary CLA on human health. In addition, some of the molecular aspects of CLA mechanisms of action have been already described, and to our knowledge, Murphy et al. [50] applied microarray technique to show the effects of CLA isomers on the global gene expression using Caco-2 cells. However, most of published results are based on studying pure, isolated isomers which may not reflect the effect of a food product naturally enriched in CLA. Thus, our research would be the first to address the effects of the extract from CLA-enriched egg yolks on a breast cancer cell model in a wide spectrum of the whole human genome.

Our previous experiments showed that EFA-CLA extract suppressed the viability of MCF-7 breast cancer cell line more effectively than extract from non-enriched egg yolks [32]. Our current study supports those findings not only for estrogen receptor-positive MCF- 7 but also for estrogen receptor-negative MDA-MB-231; however, the EFA-CLA effect was most notable for MCF-7 (Fig. 2 and Additional file 4: Figure S12), which we proposed to be associated with affected mTOR signaling pathway [8]. In addition, we also performed experiments on commercially available and described as a non-tumorigenic MCF-10A cell line (ATCC). Interestingly, we did not observe a decreased proliferation in that cell line after treatment with tested fatty acids (Fig. 3). Some authors recommend caution when using MCF-10A cell line as non-transformed human breast epithelial cells in carcinogenesis research. They point out their potential for morphological and phenotypic transformation [56]. However, it should be noted that, to some extent, this could be due to the modification of the cell line microenvironment, including culture conditions, presence of serum, and used medium [53, 76].

In the present manuscript, we discuss selected genes, which expression differs the most between cells treated with FA from CLA-enriched and non-enriched egg yolks (Table 1), specifically, in terms of their potential significance in the neoplastic process. Based on a scheme of the interactions between those genes (Fig. 5), we pointed the anti-proliferative and pro-apoptotic properties of EFA-CLA, specifically through the regulation of AKT/mTOR signaling pathway. mTOR interacts with several proteins and forms two distinct complexes named mTOR complex 1 (mTORC1) and 2 (mTORC2) of which mTORC1 is currently better characterized. mTOR is a central controller of protein synthesis, cell growth, cell proliferation, and cell viability [37]. Several components of the PI3K/PTEN/AKT/mTOR pathway (Fig. 5) are frequently mutated in human cancers.

Most notably, our results showed that treatment of MCF-7 cells with EFA-CLA (compared to EFA) increased the mRNA expression of known tumor suppressors, such as *TSC2*, *PTEN*, *PPP2R5E*, and *LMCD1*. TSC2 complex is a key upstream regulator of mTORC1, and it constitutes of two proteins, TSC1 and TSC2, that interacts with each other. Mutations in either of them result in the development of the tuberous sclerosis complex (TSC), characterized by the growth of benign tumors in multiple vital organs [29]. Reduced expression of TSC2 was determined in the invasive breast cancer compared to normal mammary epithelium [45]. Another mTOR pathway inhibitor, PTEN, was also found up-regulated. *PTEN* is a p53-regulated tumor suppressor, which transcription can be enhanced by p53 protein—acting as a transcription factor. Although our microarrays did not show statistically significant change in TP53 mRNA expression in the EFA-CLA cells vs. EFA group, additional Western blot analysis clearly showed an accumulation of p53 at the protein levels in the EFA-CLA treated cells (data not shown). *PTEN* is one of the most frequently mutated genes in various human cancers [9, 17, 70], including breast cancers, and is linked to aggressive tumors [64]. Information on *PPP2R5E* is limited, but it has been suggested as a potential negative regulator of PI3K/AKT signaling (2016). It has been also found to act as a tumor suppressor in breast cancer [19] and gastric cancer cells [38].

During our analysis, we have also found other genes potentially associated with PI3K/AKT/mTOR pathway, which were down-regulated in cells: *AGPS*, *ANXA5*, *STAT3*, *NOTCH1*, *PRKAR1A*, and *HIF1A* (Table 1), after the treatment with EFA-CLA. Whether observed decrease in gene expression is the cause or the result of inhibition of AKT/mTOR pathway requires further study. Zhu et al. [77] showed that phosphorylation of AKT1 in glioma and hepatic carcinoma cell lines was reduced simultaneously with *AGPS* silencing, whereas Benjamin et al. [6] showed that silencing of *AGPS* in breast cancer (including MCF-7) and melanoma cells manifested in a life-time reduction of cancer cells viability, tumor growth, and invasiveness. Although we did not find a direct link between *ANXA5* and AKT/mTOR pathway (Fig. 5), its up-regulation may be a predictive factor for tumor stage and clinical outcome of colorectal cancer [71]. ANXA5 has been also found in a group of pro-apoptotic genes [48]. These data may suggest that the expression of ANXA5 could be dependent on the expression profile of other superior genes being a part of anti-tumor response of cells. In our study, we show a significant down-regulation of *STAT3* and *NOTCH1* gene under the influence of EFA-CLA. Phosphorylated STAT3 is being observed in nearly 70 % of human cancers. Acting as an oncoprotein, it is constitutively activated in many primary human tumors, being activated by a number of different cytokines as well as oncoproteins, i.e., Src and Ras. NOTCH1 is associated with PI3K and PI3K-dependent activation of AKT1. It has been shown to play a role in growth, proliferation, and inhibition of apoptosis [10, 58]. It has been also reported that Hes1, NOTCH1’s downstream target protein, negatively regulates *PTEN* expression [51]. A fly model of tumorigenesis induced by NOTCH1 showed a synergism of NOTCH1 signaling and PI3K/AKT pathway, suggesting that the interplay between these two signaling pathways was conserved during the evolution process.

*PRKAR1A* has been found to be down-regulated in MCF-7 cells, due to the treatment with EFA-CLA. Information about *PRKAR1A* in available literature is ambiguous. Some studies have shown its up-regulation in many tumors, including breast cancer [7, 40, 41], suggesting its role in cell cycle regulation, growth, and/or proliferation. Other studies have pointed its tumor suppressing properties in osteosarcoma [49] and follicular thyroid cancer [55]. Due to EFA-CLA treatment, we determined a reduction in the mRNA expression of *UCP2*, which belongs to the family of mitochondrial carriers. Significant amount of studies is available on *UCP2*, but its functions are still under debate. It has been recently proposed to control routing of mitochondrial substrates [20, 69]. The overexpression of UCP2 has been shown in various tumors, including breast cancer [44]. Some data reveal that up-regulation of UCP2 may facilitate an increased chemoresistance as well as cancer adaptation to oxidative stress via mitochondrial suppression of reactive oxygen species (ROS) [4, 15, 18]. Sayeed et al. [60] have shown that *UCP2* gene silencing rapidly led to the induction of apoptosis and differentiation in breast cancer cells, concurrent with reduced cell survival and proliferation. These results may be supported by numerous studies reporting evidences suggesting a correlation between oxidative stress and breast cancer, due to mitochondrial dysfunction [57]. Being the source of ROS, mitochondria are particularly exposed to potential oxidative DNA damage. Several studies have determined a higher rate of mitochondrial DNA (mtDNA) mutations in breast tumor tissue, specifically, they identified somatic mutations in the D-loop region as, probably, the major factor leading to decreased mtDNA level in breast tumors and indicating a poor prognosis [36]. Recent results have shown that a reduced number of mtDNA copy may be involved in cancer development and/or progression [66, 75] and mtDNA content might be potentially used as a tool to predict prognosis. Interestingly, in our unpublished studies on prostate cancer cells and melanoma, we determined a significant increase in the levels of mtDNA when treating with EFA-CLA extracts (compared to EFA).

We also determined a down-regulation of *HIGD2A*, a subunit of the cytochrome C oxidase (COX, complex IV). However, little is known about its mechanism of action. A study presented by An et al. [2] on *HIGD1A*, another member of *HIG1* gene family, showed that expression of *HIGD1A* is directly dependent on binding of HIF-1α to HRE (hypoxia-response element) site at −32 bp in the *HIG1D1A* promoter. Transfecting RAW264.7 cell line with *HIGD1A* under hypoxia condition promoted cell survival, whereas silencing the endogenous gene with siRNA resulted in hypoxia-induced apoptosis. The authors have proposed the inhibition of cytochrome C release and the reduction of caspases as a potential mechanism. They also obtained similar results for *HIGD2A*. It should be noted that similarity between *HIG1* gene family members might have influenced obtained microarray results as probe specificity for gene isoforms is limited [35]. Interestingly, in our unpublished results on prostate cancer cells and melanoma, we determined a down-regulation of HIF-1α, after treatment with EFA-CLA. In our present study, we also observed down-regulation of HIF-1α in MCF-7 cells after EFA-CLA treatment, which is a positive signal confirming the anti-proliferative activity of EFA-CLA in the AKT/mTOR pathway (Fig. 5). HIF-1α is responsible for the activation of transcription of various genes, such as *VEGF*, *EDN1*, or *CDKN1A*, which are involved in cell cycle regulation, neovascularization, and metastasis [62]. Overexpression of HIF-1α correlates with an advanced tumor stage and poor survival [63, 68]. Majumder et al. [43] have demonstrated that the expansion of AKT-driven prostate epithelial cells requires mTOR-dependent survival signaling and activation of HIF-1α.

Finally, our results showed a down-regulation of *GNA12* mRNA expression after the treatment with EFA-CLA. Although we did not find a direct association with mTOR, *GNA12* has been found to participate in oncogenesis and metastasis in pathological conditions [28]. Kim et al. [31]), while studying breast cancer cells, proposed that *GNA12* up-regulates the activity of matrix metalloproteinase (MMP)-2 via p53-dependent manner and promotes malignant phenotypic conversion of this cancer cells. A recent study supported those findings [12]. Their results also show that *GNA12* stimulates the expression and activity of tumor promoting cytokines IL-6 and IL-8 and MMP-2 via binding and activation of NF-kB.

Although in current manuscript we focused on differences between the CLA-enriched (CLA-EFA) and non-enriched egg (EFAs), attached data (database GSE65397) suggest that EFAs can change the gene expression profile as well; however, they are unable to suppress the cell proliferation as efficiently as CLA-EFA can. The analysis of MCF-7 transcriptomes revealed that some of the observed changes may not be solely caused by CLA isomers present in the pool of other fatty acids identified in the enriched egg yolk. As shown in Fig. 1, the incorporation of CLA was accompanied by significant changes in the general FA profile of egg yolk. The most notable was an increase in total SFA concentration at the expense of MUFA. Our calculations showed that the altered SFA/MUFA ratio can affect the expression of some genes including *HIGD2A* and *SMS* (Additional file 12: Table S11) suggesting that the changed FA ratio in EFA-CLA extract could be responsible, at least in part, for the specific response of the cancer cells (Fig 2). It seems therefore that our functional products, obtained through the process of modification of hens’ diet, may show specific features determined by both the presence of CLA and altered SFA/MUFA ratio. This may also suggest that CLA-enriched eggs cannot be simply replaced with a synthetic CLA supplements.

Although available literature on effects of CLA on other FAs is limited, some authors have shown that treatment of cells with synthetic CLA increased the SFA/MUFA ratio in cell culture [14, 72]. As potential explanation, authors suggested that CLA could reduce the expression of SCD gene, which is responsible for conversion of SFA into MUFA. Interestingly, overexpression of SCD was associated with increased cancer cell proliferation, both in vitro for breast, prostate and lung cancer cells as well as in vivo [5, 14]. Our results showed a decreasing tendency for SCD mRNA for EFA-CLA vs. EFA treatment groups (Table 1); however, these results were statistically non-significant. Nevertheless, comparison with the negative control revealed a significant reduction in SCD expression for EFA-CLA at the level of transcription (Additional file 7: Table S5). It should be noticed, however, that the SCD mRNA levels does not necessarily correspond with this enzyme activity that has been shown by Choi et al. [14] for both MDA-MB- 231 and MCF-7 cells treated with synthetic *cis-9*, *trans-11* and *trans-10*, *cis-12* CLA isomers. *SCD* has been also reported to be involved in mTOR pathway. Scaglia and Igal [61] have showed that the down-regulation of *SCD* reduces the activity of AKT in A549 cell line (SCD-ablated A549 cells). In addition, Luyimbazi et al. [42] have observed an increase in SCD protein expression when using activators of mTOR pathway in both MCF-7 (ER+) and MDA-MB-231 (ER−) cell lines, while the use of selective mTOR inhibitors showed an opposite effect. All these data may suggest that the observed decrease in MCF-7 proliferation in the presence of EFA-CLA could result from down-regulation of AKT/mTOR signaling pathway and reduced expression of SCD and other genes involved in mTOR pathway (database GSE65397). However, the role of CLA, other egg FAs, and the altered SFA/MUFA ratio in mTOR-dependent down-regulation of cell proliferation needs further studies.

Although some of our results may require to be confirmed at protein levels, the microarrays are a valuable and multi-faceted source of information and may explain the adequacy of further in vivo research, according to 3R principles (replacement, reduction, and refinement).

## Conclusions

In summary, our study presents the first evidence that the fatty acids extracts from CLA-enriched egg yolks (EFA-CLA) can affect transcriptome of MCF-7 cancer cells and inhibit their proliferation. We found this effect to be accompanied by changes in gene expression associated with down-regulation of AKT/mTOR signaling pathway. EFA-CLA increased expression of *TSC2* and *PTEN* tumor suppressors as well as decreased the expression of oncogenes including *NOTCH1*, *AGPS*, *GNA12*, *STAT3*, *UCP2*, *HIGD2A*, *HIF1A*, and *PPKAR1A*. The observed results are most likely achieved by the combined effect of both incorporated CLA isomers and other fatty acids in eggs organically modified through hens’ diet. It seems, therefore, that in contrast to synthetic CLA supplements, CLA-enriched eggs with an altered SFA/MUFA ratio could be easily available food products with a potential of a cancer chemopreventive agent. Although this concept needs further in vivo studies, it is clear that our microarray-derived results are a rich source of information on pathways in which fatty acids from CLA-enriched egg yolks can modify the response of the cancer cells at the level of transcription.

## Abbreviations

AKT, protein kinase B; BF3, boron trifulouride; BHT, butylated hydroxytoluen; BrdU, 5′-bromo-2′-deoxy-uridine; CLA, conjugated linoleic acid; COX, cytochrome C oxidase; DNA, deoxyribonucleic acid; EC, empty control; EFA, fatty acids extract from non-enriched egg yolks; EFA-CLA, fatty acids extract from CLA-enriched egg yolks; FA, fatty acids; FAME, fatty acid methyl esters; FC, fold change; GC/MS, gas chrmoatography/mass spectrometry; GO, Gene Ontology; HRE, hypoxia-response element; KOH, potassium hydroxide; MCF-7, human breast adenocarcinoma cell line; mtDNA, mitochondrial DNA; mTOR, mammalian target of rapamycin; NC, negative control; QC, quality control; RNA, ribonucleic acid; ROS, reactive oxygen species; siRNA, small interfering RNA

## Additional files

Additional file 1: S1. Composition of hens’ experimental diets (%). (DOCX 12 kb) Additional file 2: S2. FAME analysis GC/MS conditions. (DOCX 12 kb) Additional file 3: S3. Nucleotide sequences of primers. *ACTB*, actin, beta; *CAMSAP2*, calmodulin regulated spectrin-associated protein family, member 2; *GAPDH*, glyceraldehyde-3-phosphate dehydrogenase; *HIGD2A*, HIG1 hypoxia inducible domain family, member 2A; *HPRT1*, hypoxanthine phosphoribosyltransferase 1; *HSP90AB1*, heat shock protein 90 kDa alpha (cytosolic); *NAP1L1*, nucleosome assembly protein 1-like 1; *NOTCH1*, Notch homolog 1, translocation-associated; *PPKAR1A*, protein kinase, cAMP-dependent, regulatory, type I, alpha; *PPP2R5E*,protein phosphatase 2, regulatory subunit B', epsilon isoform; *TSC2*, tuberous sclerosis 2; *UCP2* uncoupling protein 2 (mitochondrial, proton carrier). (DOCX 14 kb) Additional file 4: S12. Effect of EFA-CLA on MDA-MB-231 cells proliferation. The assay was performed using BrdU test (Roche). Values are expressed as means ± SEM for the *N* ≥ 9, standarized to NC as 100 %. Statistical significance was based on Student’s *t* test **p* < 0.05 vs. NC and ^*p* < 0.05 vs. EFA. (TIF 40 kb) Additional file 5: S4. Analysis of differently expressed transcripts between experimental groups in MCF-7 cell line. Tukey’s HSD post hoc test (*p* < 0.05); underlining determined different transcripts between the compared groups; italics determined a common transcripts between the compared groups; bold determined all the analyzed transcripts. (DOCX 12 kb) Additional file 6: S10. The list of the differently regulated EFA-CLA vs. EFA specific transcripts in MCF-7 cell line (without genes, which expression could directly be affected by the ET solvent). Statistical significance of treatment: *p* < 0.05. (DOCX 30 kb) Additional file 7: S5. The list of the differently regulated EFA vs. NC specific transcripts in MCF-7 cell line. **p* < 0.05 for EFA vs. NC; NC, negative control; NS *p* > 0.05 (DOCX 17 kb) Additional file 8: S6. Pathways based on EFA-CLA vs. EFA specific genes differently regulated in MCF-7 cell line. Statistical significance of treatment: *p* < 0.05. (DOCX 14 kb) Additional file 9: S7. Table GO biological processes based on EFA-CLA vs. EFA specific genes differently regulated in MCF-7 cell line. Statistical significance of treatment: *p* < 0.05. (DOCX 13 kb) Additional file 10: S8. GO molecular functions based on EFA-CLA vs. EFA specific genes differently regulated in MCF-7 cell line. Statistical significance of treatment: *p* < 0.05. (DOCX 13 kb) Additional file 11: S9. Protein classes based on EFA-CLA vs. EFA specific genes differently regulated in MCF-7 cell line. Statistical significance of treatment: *p* < 0.05. (DOCX 13 kb) Additional file 12: S11. Calculated effects of altered FA and SFA/MUFA (S/M) ratio on gene expression profile in MCF-7 cells. ^≠^potential role of SFA/MUFA ratio in the regulation of gene expression. Abbreviations: c9. t11CLA—cis-9.trans-11-CLA; t10. c12CLA—trans-10.cis-12-CLA. S/M. changed SFA/MUFA ratio. (DOCX 18 kb)
