# Erratum to: Fatty acid extract from CLA-enriched egg yolks can mediate transcriptome reprogramming of MCF-7 cancer cells to prevent their growth and proliferation

**DOI:** 10.1186/s12263-016-0540-4

**Published:** 2016-08-16

**Authors:** Aneta A. Koronowicz, Paula Banks, Dominik Domagała, Adam Master, Teresa Leszczyńska, Ewelina Piasna, Mariola Marynowska, Piotr Laidler

**Affiliations:** 1 Department of Human Nutrition, Faculty of Food Technology, University of Agriculture, Krakow, Poland; 2 Department of Biochemistry and Molecular Biology, Medical Centre for Postgraduate Education, Warsaw, Poland; 3 Department of Medical Biochemistry, Jagiellonian University Medical College, Krakow, Poland

## Erratum

Upon publication of the original article [1], it was noticed that the Contributions section was incorrect. This has now been corrected below in this erratum.
