# Supplementary material for: Fatty acid extract from CLA-enriched egg yolks can mediate transcriptome reprogramming of MCF-7 cancer cells to prevent their growth and proliferation
Source: Genes Nutr. 2016 Jul 27;11:22. doi: 10.1186/s12263-016-0537-z (PMC4968440; doi:10.1186/s12263-016-0537-z)
Supplement: Additional file 1: S1. — Composition of hens’ experimental diets (%). (DOCX 12 kb) [file 12263_2016_537_MOESM1_ESM.docx]

**S1 Table**

Composition of hens’ experimental diets [%]

| **Ingredient** | **Diet** | |
| --- | --- | --- |
|  | **0.00% CLA** | **0.75% CLA** |
| Wheat middling | 26.00 | 26.00 |
| Ground yellow corn | 35.00 | 35.00 |
| Soybean meal (45% CP) | 21.37 | 21.37 |
| Dried grass | 3.00 | 3.00 |
| Rapeseed oil | 1.50 | 1.50 |
| CLA^a)^ | 0.00 | 0.94 |
| Sunflower oil | 2.50 | 1.56 |
| Calcium Carbonate | 8.10 | 8.10 |
| Dicalcium phosphate | 1.70 | 1.70 |
| NaCl | 0.30 | 0.30 |
| Vitamin - mineral premix^b)^ | 0.50 | 0.50 |
| DL-methionine (99%) | 0.01 | 0.01 |
| L-lysine HCl (80%) | 0.02 | 0.02 |
| *^a)^ The source of CLA used in this experiment contained 80% of CLA (BASF, Germany)*  *^b)^Commercial premix Lutamix ‘DJ’* | | |
