# Supplementary material for: Fatty acid extract from CLA-enriched egg yolks can mediate transcriptome reprogramming of MCF-7 cancer cells to prevent their growth and proliferation
Source: Genes Nutr. 2016 Jul 27;11:22. doi: 10.1186/s12263-016-0537-z (PMC4968440; doi:10.1186/s12263-016-0537-z)
Supplement: Additional file 2: S2. — FAME analysis GC/MS conditions. (DOCX 12 kb) [file 12263_2016_537_MOESM2_ESM.docx]

**S2 Table**

FAME analysis GC/MS conditions

| Parameter | Setting |
| --- | --- |
| GC/MS System | Simadzu (QP 5050A) |
| Analytical column | SP™-2560 (100m x 0·25mm; 0·25 µm) |
| Injector Temperature | 245°C |
| Injection volume | 1μL |
| Carrier Gas | Helium & 1.8mL/min |
| Oven | 60°C (5 min), 15°C/min to 180°C (16 min), 5°C/min to 220°C (7 min) |
| Detector | FID, 245^O^C |
| Ionization mode | EI |
| Voltage | 70eV |
| Function type | Full Scan |
| Scan range | *m/z* 40-500 |
| Identification reference | FAMEMix (LarodanFine Chemicals) and mass spectralibrary(NIST1.7) |
