# Supplementary material for: Fatty acid extract from CLA-enriched egg yolks can mediate transcriptome reprogramming of MCF-7 cancer cells to prevent their growth and proliferation
Source: Genes Nutr. 2016 Jul 27;11:22. doi: 10.1186/s12263-016-0537-z (PMC4968440; doi:10.1186/s12263-016-0537-z)
Supplement: Additional file 3: S3. — Nucleotide sequences of primers. ACTB, actin, beta; CAMSAP2, calmodulin regulated spectrin-associated protein family, member 2; GAPDH, glyceraldehyde-3-phosphate dehydrogenase; HIGD2A, HIG1 hypoxia inducible domain family, member 2A; HPRT1, hypoxanthine phosphoribosyltransferase 1; HSP90AB1, heat shock protein 90 kDa alpha (cytosolic); NAP1L1, nucleosome assembly protein 1-like 1; NOTCH1, Notch homolog 1, translocation-associated; PPKAR1A, protein kinase, cAMP-dependent, regulatory, type I, alpha; PPP2R5E,protein phosphatase 2, regulatory subunit B', epsilon isoform; TSC2, tuberous sclerosis 2; UCP2 uncoupling protein 2 (mitochondrial, proton carrier). (DOCX 14 kb) [file 12263_2016_537_MOESM3_ESM.docx]

**S3 Table**

Nucleotide sequences of primers

| Gene | Primers forward 5’→3’ | Primers reverse 5’→3’ | Reference |
| --- | --- | --- | --- |
| *CAMSAP2* | GGAAACTGGTTCCAGCTCGTTA | CAGCCATCTGTCCCATCCTT | NM_001297707.1 |
| *HIGD2A* | TGGTACCCATAGGTTGCCTG | TGCGCATCATGAGCTGAGAG | NM_138820.2 |
| *NAP1L1* | TTTTTAGCGCCATCTGCTCG | AGAACTCCAAATATCGGCGAC | NM_139207.2 |
| *NOTCH1* | GACAGCCTCAACGGGTACAA | CACACGTAGCCACTGGTCAT | NM_017617.3 |
| *PPKAR1A* | CTGGACAAGTGGGAACGTCT | ACAGCAGCTGACCCCTCTA | NM_001278433.1 |
| *PPP2R5E* | CTGGACAAGTGGGAACGTCT | ACAGCAGCTGACCCCTCTA | NM_001278433.1 |
| *TSC2* | TACAGACGTCCCTCACCAGT | AGGAGACCTCTTCGGGACAG | NM_000548.3 |
| *UCP2* | ACCTCTCCCAATGTTGCTCG | GCAAGGGAGGTCATCTGTCA | NM_003355.2 |
| *ACTB* | GCTGGGGTGTTGAAGGTCTC | CGGCATCGTCACCAACTG | NM_001101.3 |
| *GAPDH* | ACCATCTTCCAGGAGCGAGA | GACTCCACGACGTACTCAGC | NM_001289746.1 |
| *HPRT1* | CAGAGGGCTACAATGTGATG | TGGCGTCGTGATTAGTGATG | NM_000194.2 |
| *HSP90AB1* | CCACTTGGCAGTCAAGCACT | CACACGGCGGACATAGAGTT | NM_001271972.1 |

### *ACTB,* actin, beta; *CAMSAP2,* calmodulin regulated spectrin-associated protein family, member 2; *GAPDH,* glyceraldehyde-3-phosphate dehydrogenase; *HIGD2A,* HIG1 hypoxia inducible domain family, member 2A; *HPRT1,* hypoxanthine phosphoribosyltransferase 1; *HSP90AB1*, heat shock protein 90kDa alpha (cytosolic); *NAP1L1*, nucleosome assembly protein 1-like 1; *NOTCH1,* Notch Homolog 1, Translocation-Associated; *PPKAR1A,* protein kinase, cAMP-dependent, regulatory, type I, alpha; *PPP2R5E,*protein phosphatase 2, regulatory subunit B', epsilon isoform; *TSC2,* tuberous sclerosis 2; *UCP2* Uncoupling Protein 2 (Mitochondrial, Proton Carrier).
