# Supplementary figures and images for: Fatty acid extract from CLA-enriched egg yolks can mediate transcriptome reprogramming of MCF-7 cancer cells to prevent their growth and proliferation
Source: Genes Nutr. 2016 Jul 27;11:22. doi: 10.1186/s12263-016-0537-z (PMC4968440; doi:10.1186/s12263-016-0537-z)

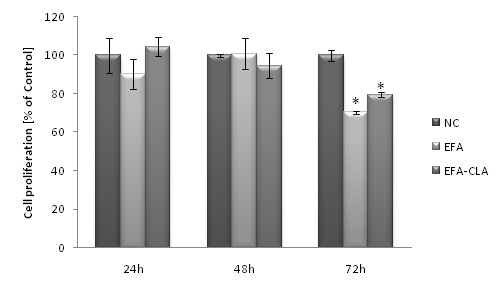

Supplement: Additional file 4: S12. — Effect of EFA-CLA on MDA-MB-231 cells proliferation. The assay was performed using BrdU test (Roche). Values are expressed as means ± SEM for the N ≥ 9, standarized to NC as 100 %. Statistical significance was based on Student’s t test *p < 0.05 vs. NC and ^p < 0.05 vs. EFA. (TIF 40 kb) [file 12263_2016_537_MOESM4_ESM.tif]
