# Supplementary material for: Fatty acid extract from CLA-enriched egg yolks can mediate transcriptome reprogramming of MCF-7 cancer cells to prevent their growth and proliferation
Source: Genes Nutr. 2016 Jul 27;11:22. doi: 10.1186/s12263-016-0537-z (PMC4968440; doi:10.1186/s12263-016-0537-z)
Supplement: Additional file 5: S4. — Analysis of differently expressed transcripts between experimental groups in MCF-7 cell line. Tukey’s HSD post hoc test (p < 0.05); underlining determined different transcripts between the compared groups; italics determined a common transcripts between the compared groups; bold determined all the analyzed transcripts. (DOCX 12 kb) [file 12263_2016_537_MOESM5_ESM.docx]

**S4 Table**

Analysis of differently expressed transcripts between experimental groups in MCF-7 cell line

| Grupe Name | EFA | EFA-CLA | ET | NC |
| --- | --- | --- | --- | --- |
| EFA | **1589** | 160 | 1447 | 1508 |
| EFA-CLA | *1438* | **1589** | 1404 | 1497 |
| ET | *151* | *194* | **1589** | 131 |
| NC | *90* | *101* | *1467* | **1589** |

Tukey's HSD Post-hoc test (p < 0.05)

Underlining determined different transcripts between the compared groups

*Italics* determined a common transcripts between the compared groups

**Bold** determined all the analyzed transcripts
