# Supplementary material for: Fatty acid extract from CLA-enriched egg yolks can mediate transcriptome reprogramming of MCF-7 cancer cells to prevent their growth and proliferation
Source: Genes Nutr. 2016 Jul 27;11:22. doi: 10.1186/s12263-016-0537-z (PMC4968440; doi:10.1186/s12263-016-0537-z)
Supplement: Additional file 6: S10. — The list of the differently regulated EFA-CLA vs. EFA specific transcripts in MCF-7 cell line (without genes, which expression could directly be affected by the ET solvent). Statistical significance of treatment: p < 0.05. (DOCX 30 kb) [file 12263_2016_537_MOESM6_ESM.docx]

**S10 Table**

The list of the differently regulated EFA-CLA vs. EFA specific transcripts in MCF-7 cell line (without genes, which expression could directly be affected by the ET solvent)

| **Gene Symbol** | **Adjusted p-values** | **FC value** | **Gene Name** |
| --- | --- | --- | --- |
| *NOTCH1* | 0.0146 | -2.6300 | Notch Homolog 1, Translocation-Associated |
| *XLOC_000385* | 0.0020 | -2.0228 | BROAD Institute lincRNA ] |
| *Uncharacterized* | 0.0000 | -1.9050 | Uncharacterized |
| *SLC9A1* | 0.0017 | -1.8525 | Solute carrier family 9, subfamily A (NHE1, cation proton antiporter 1), member 1 |
| *HMOX1* | 0.0006 | -1.8137 | Heme oxygenase (decycling) 1 |
| *C1QTNF9B-AS1* | 0.0002 | -1.6872 | C1QTNF9B antisense RNA 1 |
| *CEACAM1* | 0.0001 | -1.6304 | Carcinoembryonic antigen-related cell adhesion molecule 1 (biliary glycoprotein) |
| *HIF1A* | 0.0184 | -1.56 | Hypoxia Inducible Factor 1, Alpha Subunit (Basic Helix-Loop-Helix Transcription Factor) |
| *Uncharacterized* | 0.0004 | -1.4986 | Uncharacterized |
| *GNA12* | 0.0006 | -1.4795 | Guanine nucleotide binding protein (G protein) alpha 12 |
| *CHRNA7* | 0.0001 | -1.4716 | Cholinergic receptor, nicotinic, alpha 7 (neuronal) |
| *AGPS* | 0.0017 | -1.4524 | Alkylglycerone phosphate synthase |
| *C1orf52* | 0.0010 | -1.3950 | Chromosome 1 open reading frame 52 |
| *MYRIP* | 0.0000 | -1.3010 | Myosin VIIA and Rab interacting protein |
| *UCP2* | 0.0001 | -1.2914 | Uncoupling protein 2 (mitochondrial, proton carrier) |
| *WASH1* | 0.0015 | -1.2694 | WAS protein family homolog 1 |
| *HIGD2A* | 0.0002 | -1.2679 | HIG1 hypoxia inducible domain family, member 2A |
| *BLCAP* | 0.0021 | -1.2563 | Bladder cancer associated protein |
| *LCP1* | 0.0006 | -1.2426 | Lymphocyte cytosolic protein 1 (L-plastin) |
| *MAT2A* | 0.0022 | -1.2414 | Methionine adenosyltransferase II, alpha |
| *S100P* | 0.0000 | -1.2394 | S100 calcium binding protein P |
| *GTPBP2* | 0.0005 | -1.2322 | GTP binding protein 2 |
| *MT1B* | 0.0000 | -1.2270 | Metallothionein 1B |
| *ANG* | 0.0000 | -1.2194 | Angiogenin, ribonuclease, RNase A family, 5 |
| *IRF7* | 0.0001 | -1.2190 | Interferon regulatory factor 7 |
| *MT1E* | 0.0000 | -1.2170 | Metallothionein 1E |
| *FTH1* | 0.0000 | -1.2162 | Ferritin, heavy polypeptide 1 |
| *AKR1C3* | 0.0000 | -1.2122 | Aldo-keto reductase family 1, member C3 |
| *NDRG1* | 0.0000 | -1.2104 | N-myc downstream regulated 1 |
| *ALG3* | 0.0003 | -1.2008 | ALG3, alpha-1,3- mannosyltransferase |
| *RAB7L1* | 0.0014 | -1.1942 | RAB7, member RAS oncogene family-like 1 |
| *FTH1* | 0.0000 | -1.1909 | Ferritin, heavy polypeptide 1 |
| *DHRS3* | 0.0000 | -1.1883 | Dehydrogenase/reductase (SDR family) member 3 |
| *HSP90B1* | 0.0005 | -1.1875 | Heat shock protein 90kDa beta (Grp94), member 1 |
| *KIAA0513* | 0.0000 | -1.1854 | KIAA0513 |
| *CRABP2* | 0.0000 | -1.1747 | Cellular retinoic acid binding protein 2 |
| *SQSTM1* | 0.0000 | -1.1736 | Sequestosome 1 |
| *BIN3* | 0.0021 | -1.1640 | Bridging integrator 3 |
| *RTN4R* | 0.0021 | -1.1614 | Reticulon 4 receptor |
| *NEU1* | 0.0000 | -1.1607 | Sialidase 1 (lysosomal sialidase) |
| *BSPRY* | 0.0001 | -1.1583 | B-box and SPRY domain containing |
| *LOC392288* | 0.0006 | -1.1487 | Microtubule-associated proteins 1A/1B light chain 3B-like\ |
| *SERPINA3* | 0.0000 | -1.1450 | Serpin peptidase inhibitor, clade A (alpha-1 antiproteinase, antitrypsin), member 3 |
| *PRKAR1A* | 0.0020 | -1.1435 | Protein kinase, cAMP-dependent, regulatory, type I, alpha |
| *SKA2* | 0.0012 | -1.1429 | Spindle and kinetochore associated complex subunit 2 |
| *HLA-J* | 0.0000 | -1.1356 | Major histocompatibility complex, class I, J (pseudogene) |
| *TNFRSF21* | 0.0000 | -1.1342 | Tumor necrosis factor receptor superfamily, member 21 |
| *STAT5B* | 0.0021 | -1.1339 | Signal transducer and activator of transcription 5B |
| *NDUFB11* | 0.0002 | -1.1336 | NADH dehydrogenase (ubiquinone) 1 beta subcomplex, 11, 17.3kDa |
| *ACTB* | 0.0014 | -1.1318 | Actin, beta |
| *STAT3* | 0.0088 | -1.3200 | Signal Transducer And Activator Of Transcription 3 (Acute-Phase Response Factor) |
| *MT1X* | 0.0000 | -1.1310 | metallothionein 1X |
| *FAM213B* | 0.0001 | -1.1188 | Family with sequence similarity 213, member B |
| *LOC388152* | 0.0002 | -1.1124 | Golgin A2 pseudogene |
| *PPP2R2A* | 0.0001 | -1.1071 | Protein phosphatase 2, regulatory subunit B, alpha |
| *PELI3* | 0.0015 | -1.1068 | Pellino E3 ubiquitin protein ligase family member 3 |
| *GSR* | 0.0001 | -1.1027 | Glutathione reductase |
| *TMEM189* | 0.0010 | -1.1003 | Transmembrane protein 189 |
| *PITRM1* | 0.0008 | -1.0928 | Pitrilysin metallopeptidase 1 |
| *XLOC_014512* | 0.0001 | -1.0893 | BROAD Institute lincRNA |
| *ANXA5* | 0.0011 | -1.0489 | Annexin A5 |
| *SUMO2* | 0.0000 | 1.0512 | Small ubiquitin-like modifier 2 |
| *PRIM2* | 0.0001 | 1.0774 | Primase, DNA, polypeptide 2 (58kDa) |
| *SMS* | 0.0016 | 1.0830 | Spermine synthase |
| *RIOK2* | 0.0001 | 1.0859 | RIO kinase 2 |
| *PWP1* | 0.0019 | 1.0862 | PWP1 homolog (S. cerevisiae) |
| *SLC39A8* | 0.0000 | 1.0896 | Solute carrier family 39 (zinc transporter), member 8 |
| *USP18* | 0.0020 | 1.0954 | Ubiquitin specific peptidase 18 |
| *DCLRE1C* | 0.0000 | 1.0976 | DNA cross-link repair 1C |
| *VCPIP1* | 0.0011 | 1.1025 | Valosin containing protein (p97)/p47 complex interacting protein 1 |
| *MSH2* | 0.0018 | 1.1134 | MutS homolog 2 |
| *NAE1* | 0.0017 | 1.1138 | NEDD8 activating enzyme E1 subunit 1 |
| *LOC101926980* | 0.0001 | 1.1222 | Uncharacterized LOC101926980 |
| *NASP* | 0.0001 | 1.1244 | Nuclear autoantigenic sperm protein (histone-binding) |
| *PAFAH1B1* | 0.0001 | 1.1255 | Platelet-activating factor acetylhydrolase 1b, regulatory subunit 1 (45kDa) |
| *FAM120C* | 0.0000 | 1.1263 | Family with sequence similarity 120C |
| *NASP* | 0.0002 | 1.1271 | Nuclear autoantigenic sperm protein (histone-binding) |
| *MSX2P1* | 0.0000 | 1.1284 | Msh homeobox 2 pseudogene 1 |
| *ALMS1* | 0.0000 | 1.1320 | Alstrom syndrome 1 |
| *PPP2R5E* | 0.0003 | 1.1344 | Protein phosphatase 2, regulatory subunit B', epsilon isoform |
| *KIAA0101* | 0.0000 | 1.1367 | KIAA0101 |
| *CRBN* | 0.0016 | 1.1376 | Cereblon |
| *BLOC1S5* | 0.0000 | 1.1430 | Biogenesis of lysosomal organelles complex-1, subunit 5, muted |
| *CBWD5* | 0.0000 | 1.1460 | COBW domain containing 5 |
| *PTEN* | 0.0382 | 1.15 | Phosphatidylinositol 3,4,5-Trisphosphate 3-Phosphatase And Dual-Specificity Protein Phosphatase PTEN |
| *SMN1* | 0.0004 | 1.1483 | Survival of motor neuron 1, telomeric |
| *STRAP* | 0.0005 | 1.1515 | Serine/threonine kinase receptor associated protein |
| *SREBF1* | 0.0003 | 1.1529 | Sterol regulatory element binding transcription factor 1 |
| *TBC1D9* | 0.0000 | 1.1564 | TBC1 domain family, member 9 (with GRAM domain) |
| *GSTM1* | 0.0000 | 1.1597 | Glutathione S-transferase mu 1 |
| *OXR1* | 0.0000 | 1.1598 | Oxidation resistance 1 |
| *HNRNPA3* | 0.0002 | 1.1650 | Heterogeneous nuclear ribonucleoprotein A3 |
| *CDK6* | 0.0000 | 1.1674 | Cyclin-dependent kinase 6 |
| *NAP1L1* | 0.0012 | 1.1731 | Nucleosome assembly protein 1-like 1 |
| *SERTAD4* | 0.0005 | 1.1739 | SERTA domain containing 4 |
| *LOC646214* | 0.0006 | 1.1792 | P21 protein (Cdc42/Rac)-activated kinase 2 pseudogene |
| *C12orf76* | 0.0000 | 1.1798 | Chromosome 12 open reading frame 76 |
| *BAG3* | 0.0000 | 1.1814 | BCL2-associated athanogene 3 |
| *TRPS1* | 0.0004 | 1.1841 | Trichorhinophalangeal syndrome I |
| *GFRA1* | 0.0000 | 1.1863 | GDNF family receptor alpha 1 |
| *RASA3* | 0.0002 | 1.1894 | RAS p21 protein activator 3 |
| *MTHFD2* | 0.0000 | 1.1913 | Methylenetetrahydrofolate dehydrogenase (NADP+ dependent) 2, methenyltetrahydrofolate cyclohydrolase |
| *GLIS2* | 0.0007 | 1.1927 | GLIS family zinc finger 2 |
| *RAB39B* | 0.0015 | 1.2058 | RAB39B, member RAS oncogene family |
| *LMCD1* | 0.0001 | 1.2108 | LIM and cysteine-rich domains 1 |
| *PLCB1* | 0.0000 | 1.2166 | Phospholipase C, beta 1 (phosphoinositide-specific) |
| *CA5B* | 0.0001 | 1.2186 | Carbonic anhydrase VB, mitochondrial |
| *MYB* | 0.0000 | 1.2198 | V-myb avian myeloblastosis viral oncogene homolog |
| *PTPN14* | 0.0000 | 1.2229 | Protein tyrosine phosphatase, non-receptor type 14 |
| *ARL4A* | 0.0001 | 1.2257 | ADP-ribosylation factor-like 4A |
| *TSC2* | 0.0008 | 1.2298 | Tuberous sclerosis 2 |
| *CAMSAP2* | 0.0004 | 1.2324 | Calmodulin regulated spectrin-associated protein family, member 2 |
| *CAMK2N1* | 0.0013 | 1.2378 | Calcium/calmodulin-dependent protein kinase II inhibitor 1 |
| *MYLIP* | 0.0006 | 1.2394 | Myosin regulatory light chain interacting protein |
| *PTMA* | 0.0005 | 1.2416 | Prothymosin, alpha |
| *TUBB3* | 0.0000 | 1.2468 | Tubulin, beta 3 class III |
| *GSTM3* | 0.0000 | 1.2548 | Glutathione S-transferase mu 3 (brain) |
| *PPP1R3C* | 0.0004 | 1.2631 | Protein phosphatase 1, regulatory subunit 3C |
| *TP53INP1* | 0.0019 | 1.2716 | Tumor protein p53 inducible nuclear protein 1 |
| *FANCD2* | 0.0003 | 1.2725 | Fanconi anemia, complementation group D2 |
| *SMC1A* | 0.0005 | 1.2761 | Structural maintenance of chromosomes 1A |
| *FLJ45139* | 0.0016 | 1.3319 | FLJ45139 protein |
| *PPM1K* | 0.0002 | 1.3405 | Protein phosphatase, Mg2+/Mn2+ dependent, 1K |
| *XLOC_000832* | 0.0022 | 1.3814 | BROAD Institute lincRNA |
| *PGR* | 0.0000 | 1.3888 | Progesterone receptor |
| *XLOC_011725* | 0.0019 | 1.4599 | BROAD Institute lincRNA |
| *LOC101927389* | 0.0009 | 1.4638 | Uncharacterized LOC101927389 |
| *LOC100131532* | 0.0018 | 1.5003 | Uncharacterized LOC100131532 |
| *PCDH9* | 0.0000 | 1.5488 | Protocadherin 9 |
| *Uncharacterized* | 0.0007 | 1.6353 | Uncharacterized |
| *LINC00992* | 0.0000 | 1.6465 | Long intergenic non-protein coding RNA 992 |
| *LINC00839* | 0.0015 | 1.6476 | Long intergenic non-protein coding RNA 839 |
| *CHSY3* | 0.0016 | 1.7184 | Chondroitin sulfate synthase 3 |
| *OVOS* | 0.0016 | 1.7184 | Ovostatin |
| *Uncharacterized* | 0.0021 | 1.7269 | Uncharacterized |
| *LOC101928550* | 0.0014 | 1.7417 | PREDICTED: Homo sapiens uncharacterized LOC101928550 |
| *Uncharacterized* | 0.0012 | 1.7493 | Uncharacterized |
| *XLOC_014512* | 0.0011 | 1.7851 | BROAD Institute lincRNA |
| *Uncharacterized* | 0.0012 | 1.8100 | Uncharacterized |
| *Uncharacterized* | 0.0003 | 2.0763 | Uncharacterized |

Statistical significance of treatment: p < 0.05
