# Supplementary material for: Fatty acid extract from CLA-enriched egg yolks can mediate transcriptome reprogramming of MCF-7 cancer cells to prevent their growth and proliferation
Source: Genes Nutr. 2016 Jul 27;11:22. doi: 10.1186/s12263-016-0537-z (PMC4968440; doi:10.1186/s12263-016-0537-z)
Supplement: Additional file 7: S5. — The list of the differently regulated EFA vs. NC specific transcripts in MCF-7 cell line. *p < 0.05 for EFA vs. NC; NC, negative control; NS p > 0.05 (DOCX 17 kb) [file 12263_2016_537_MOESM7_ESM.docx]

**S5 Table**

The list of the differently regulated EFA vs. NC specific transcripts in MCF-7 cell line

| **Gene Symbol** | **Adjusted p-values**  **EFA-CLA vs. EFA** | **FC value**  **EFA-CLA vs. EFA** | FC value EFA vs.NC | **Gene Name** |
| --- | --- | --- | --- | --- |
| ***NOTCH1*** | **0.0146** | **-2.63** | 1.17^NS^ | Notch Homolog 1, Translocation-Associated |
| ***AGPS*** | **0.0424** | **-2.19** | -1.74^NS^ | Alkylglycerone phosphate synthase |
| ***GNA12*** | **0.0009** | **-1.56** | 1.10^NS^ | Guanine nucleotide binding protein (G protein) alpha 12 |
| ***HIF1A*** | **0.0184** | **-1.56** | 1.09^NS^ | Hypoxia Inducible Factor 1, Alpha Subunit (Basic Helix-Loop-Helix Transcription Factor) |
| ***STAT3*** | **0.0088** | **-1.32** | -1.04^NS^ | Signal Transducer And Activator Of Transcription 3 (Acute-Phase Response Factor) |
| ***UCP2*** | **0.0083** | **-1.29** | -1.11^NS^ | Uncoupling protein 2 (mitochondrial, proton carrier) |
| ***HIGD2A*** | **0.0089** | **-1.27** | -1.06^NS^ | HIG1 hypoxia inducible domain family, member 2A |
| ***WASH1*** | **0.0038** | **-1.27** | -1.20^NS^ | WAS protein family homolog 1 |
| ***BIN3*** | **0.0477** | **-1.16** | -1.09^NS^ | Bridging integrator 3 |
| ***PRKAR1A*** | **0.0462** | **-1.14** | -1.03^NS^ | Protein kinase, cAMP-dependent, regulatory, type I, alpha |
| ***NDUFB11*** | **0.0105** | **-1.13** | -1.09^NS^ | NADH dehydrogenase (ubiquinone) 1 beta subcomplex, 11, 17.3kDa |
| ***ANXA5*** | **0.0311** | **-1.05** | -1.02^NS^ | Annexin A5 |
| ***SMS*** | **0.0399** | **1.08** | 1.02^NS^ | Spermine synthase |
| ***PPP2R5E*** | **0.0009** | **1.13** | 1.08^NS^ | Protein phosphatase 2, regulatory subunit B', epsilon isoform |
| ***NAP1L1*** | **0.0166** | **1.14** | 1.16^NS^ | Nucleosome assembly protein 1-like 1 |
| ***PTEN*** | **0.0382** | **1.15** | -1.02^NS^ | Phosphatidylinositol 3,4,5-Trisphosphate 3-Phosphatase And Dual-Specificity Protein Phosphatase PTEN |
| ***LOC646214*** | **0.0213** | **1.16** | 1.00^NS^ | p21 protein (Cdc42/Rac)-activated kinase 2 pseudogene |
| ***LMCD1*** | **0.0067** | **1.21** | 1.19^NS^ | LIM and cysteine-rich domains 1 |
| ***CAMSAP2*** | **0.0174** | **1.23** | 1.09^NS^ | Calmodulin regulated spectrin-associated protein family, member 2 |
| ***TSC2*** | **0.0177** | **1.26** | -1.37^NS^ | Tuberous sclerosis 2 |
| ***FLJ45139*** | **0.0406** | **1.33** | 1.36^NS^ | FLJ45139 protein |
| ***CHSY3*** | **0.0399** | **1.72** | 1.17^NS^ | Chondroitin sulfate synthase 3 |
| ***OVOS*** | **0.0403** | **1.72** | -1.10^NS^ | Ovostatin |
| ***SCD*** | **0,19** | **-1.18** | -1.30******* | Stearoyl-CoA Desaturase (Delta-9-Desaturase) |

* P<0,05 for EFA vs. NC; NC, negative control; NS P >0.05
