# Supplementary material for: Fatty acid extract from CLA-enriched egg yolks can mediate transcriptome reprogramming of MCF-7 cancer cells to prevent their growth and proliferation
Source: Genes Nutr. 2016 Jul 27;11:22. doi: 10.1186/s12263-016-0537-z (PMC4968440; doi:10.1186/s12263-016-0537-z)
Supplement: Additional file 8: S6. — Pathways based on EFA-CLA vs. EFA specific genes differently regulated in MCF-7 cell line. Statistical significance of treatment: p < 0.05. (DOCX 14 kb) [file 12263_2016_537_MOESM8_ESM.docx]

**S6 Table**

Pathways based on EFA-CLA vs. EFA specific genes differently regulated in MCF-7 cell line

| Pathway | The number of   involved genes | The symbol of | *p*-value |
| --- | --- | --- | --- |
|  |  | regulated gen |  |
| Angiogenesis | 154 | *HIF1A, HIGD2A, NOTCH1, STAT3* | 4.20E-04 |
| Insulin/IGF pathway-protein kinase B signaling cascade | 39 | *PTEN, TSC2* | 5.88E-04 |
| EGF receptor signaling pathway | 130 | *STAT3, PPP2R5E* | 6.22E-03 |
| CCKR signaling map | 169 | *PTEN, STAT3* | 1.03E-02 |
| JAK/STAT signaling pathway | 18 | *STAT3* | 1.63E-02 |
| Gonadotropin releasing hormone receptor pathway | 225 | *ANAX5A, STAT3* | 1.77E-02 |
| Hedgehog signaling pathway | 22 | *PRKAR1A* | 1.99E-02 |
| Inflammation mediated by chemokine and cytokine signaling pathway | 245 | *PTEN, STAT3* | 2.07E-02 |
| p53 pathway by glucose deprivation | 24 | *TSC2* | 2.17E-02 |
| Metabotropic glutamate receptor group I pathway | 32 | *PRKAR1A* | 2.88E-02 |
| Metabotropic glutamate receptor group II pathway | 34 | *PRKAR1A* | 3.06E-02 |
| GABA-B_receptor_II_signaling | 38 | *PRKAR1A* | 3.41E-02 |
| Notch signaling pathway | 41 | *NOTCH1* | 3.68E-02 |
| Hypoxia response via HIF activation | 28 | *HIF1A, PTEN* | 3.38E-04 |
| PI3 kinase pathway | 49 | *PTEN* | 4.38E-02 |
| Muscarinic acetylcholine receptor 2 and 4 signaling pathway | 49 | *PRKAR1A* | 4.38E-02 |
| Transcription regulation by bZIP transcription factor | 51 | *PRKAR1A* | 4.55E-02 |
| p53 pathway feedback loops 2 | 51 | *PTEN* | 4.55E-02 |

Statistical significance of treatment: p < 0.05
