# Supplementary material for: Fatty acid extract from CLA-enriched egg yolks can mediate transcriptome reprogramming of MCF-7 cancer cells to prevent their growth and proliferation
Source: Genes Nutr. 2016 Jul 27;11:22. doi: 10.1186/s12263-016-0537-z (PMC4968440; doi:10.1186/s12263-016-0537-z)
Supplement: Additional file 9: S7. — Table GO biological processes based on EFA-CLA vs. EFA specific genes differently regulated in MCF-7 cell line. Statistical significance of treatment: p < 0.05. (DOCX 13 kb) [file 12263_2016_537_MOESM9_ESM.docx]

**S7 Table**

GO biological processes based on EFA-CLA vs. EFA specific genes differently regulated in MCF-7 cell line

| Biological Process | The number of involved genes | The number of | *p*-value |
| --- | --- | --- | --- |
|  |  | regulated genes |  |
| Regulation of phosphate metabolic process | 67 | 2 | 1.10E-03 |
| Matabolic process | 8613 | 12 | 4.33E-03 |
| Catabolic process | 512 | 2 | 5.82E-03 |
| Phosphate-containing compound metabolic process | 520 | 3 | 6.02E-03 |
| Regulation of catalytic activity | 1119 | 4 | 7.67E-03 |
| Regulation of molecular function | 1140 | 4 | 8.19E-03 |
| Cellular protein modification process | 1204 | 4 | 9.90E-03 |
| Cellular process | 5952 | 9 | 1.37E-02 |
| Primary metabolic process | 7177 | 10 | 1.45E-02 |
| Nitrogen compound metabolic process | 308 | 2 | 2.10E-02 |
| Lipid metabolic process | 902 | 3 | 2.65E-02 |
| Metabolic process | 8247 | 13 | 1.06E-02 |

Statistical significance of treatment: p < 0.05
