# Supplementary material for: Fatty acid extract from CLA-enriched egg yolks can mediate transcriptome reprogramming of MCF-7 cancer cells to prevent their growth and proliferation
Source: Genes Nutr. 2016 Jul 27;11:22. doi: 10.1186/s12263-016-0537-z (PMC4968440; doi:10.1186/s12263-016-0537-z)
Supplement: Additional file 10: S8. — GO molecular functions based on EFA-CLA vs. EFA specific genes differently regulated in MCF-7 cell line. Statistical significance of treatment: p < 0.05. (DOCX 13 kb) [file 12263_2016_537_MOESM10_ESM.docx]

**S8 Table**

GO molecular functions based on EFA-CLA vs. EFA specific genes differently regulated in MCF-7 cell line

| Molecular Function | The number of  involved genes | The number of | *p*-value |
| --- | --- | --- | --- |
|  |  | regulated genes |  |
| Catalytic activity | 5529 | 10 | 1.85E-03 |
| Pyrophosphatase activity | 271 | 2 | 1.65E-02 |
| Phosphatase activity | 300 | 2 | 2.00E-02 |
| Phosphatase inhibitor activity | 36 | 1 | 2.61E-02 |
| Transferase activity | 1614 | 4 | 2.66E-02 |
| Enzyme regulator activity | 1091 | 3 | 4.30E-02 |
| Adenylate cyclase activity | 67 | 1 | 4.80E-02 |
| Protein binding | 2855 | 5 | 4.84E-02 |
| Enzyme regulator activity | 1091 | 3 | 4.30E-02 |
| Phosphoproteinphosphataseactivity | 196 | 2 | 1.36E-02 |
| Hydrolase activity. acting on ester bonds | 681 | 3 | 2.29E-02 |
| Hydrolaseactivity | 2205 | 5 | 4.37E-02 |

Statistical significance of treatment: p < 0.05
