# Supplementary material for: Fatty acid extract from CLA-enriched egg yolks can mediate transcriptome reprogramming of MCF-7 cancer cells to prevent their growth and proliferation
Source: Genes Nutr. 2016 Jul 27;11:22. doi: 10.1186/s12263-016-0537-z (PMC4968440; doi:10.1186/s12263-016-0537-z)
Supplement: Additional file 11: S9. — Protein classes based on EFA-CLA vs. EFA specific genes differently regulated in MCF-7 cell line. Statistical significance of treatment: p < 0.05. (DOCX 13 kb) [file 12263_2016_537_MOESM11_ESM.docx]

**S9 Table**

Protein classes based on EFA-CLA vs. EFA specific genes differently regulated in MCF-7 cell line

| Protein Class | The number of  involved genes | The symbol of  regulated genes | *P*-value |
| --- | --- | --- | --- |
|  |  |  |  |
| Enzyme modulator | 1439 | *NAP1L1, PRKAR1A, GNA12, TSC2* | 1.82E-02 |
| Phosphatase inhibitor | 36 | *NAP1L1* | 2.61E-02 |
| Heterotrimeric G-protein | 43 | *GNA12* | 3.11E-02 |
| Transfer/carrier protein | 439 | *UCP2* | 4.03E-02 |
| Mitochondrialcarrier protein | 62 | *UCP2* | 4.45E-02 |
| Phosphatase modulator | 56 | *NAP1L1* | 4.99E-02 |
| Protein phosphatase | 187 | *PTEN, PPP2R5E* | 1.25E-02 |
| Phosphatase | 307 | *PTEN, PPP2R5E* | 3.15E-02 |

Statistical significance of treatment: p < 0.05
