# Supplementary material for: Fatty acid extract from CLA-enriched egg yolks can mediate transcriptome reprogramming of MCF-7 cancer cells to prevent their growth and proliferation
Source: Genes Nutr. 2016 Jul 27;11:22. doi: 10.1186/s12263-016-0537-z (PMC4968440; doi:10.1186/s12263-016-0537-z)
Supplement: Additional file 12: S11. — Calculated effects of altered FA and SFA/MUFA (S/M) ratio on gene expression profile in MCF-7 cells. ≠potential role of SFA/MUFA ratio in the regulation of gene expression. Abbreviations: c9. t11CLA—cis-9.trans-11-CLA; t10. c12CLA—trans-10.cis-12-CLA. S/M. changed SFA/MUFA ratio. (DOCX 18 kb) [file 12263_2016_537_MOESM12_ESM.docx]

**S11 Table**

Calculated effects of altered FA and SFA/MUFA (S/M) ratio on gene-expression profile in MCF-7 cells.

| 1 | 2 | 3 | 4 | 5 | 6 | 7 | 8 |
| --- | --- | --- | --- | --- | --- | --- | --- |
| FC value | EFA-CLA vs. EFA | EFA vs. NC | EFA+c9 vs. EFA | EFA+t10 vs. EFA | EFA-CLA vs. EFA+c9 | EFA-CLA vs. EFA+t10 | S/M  - average of 6/5 and 7/4 |
| Gene ↓ | (~CLA+S/M) | (~EFA) | (~c9) | (~t10) | (~S/M+t10) | (~S/M+c9) | (**S/M**) |
| *NOTCH1* | -2.63 | 1.17 | -1.38 | -1.79 | -1.91 | -1.47 | -1.07 |
| *AGPS* | -2.19 | -1.74 | -1.28 | -2.96 | -1.96 | 1.18 | 1.51 |
| *GNA12* | -1.56 | 1.10 | -1.35 | -1.14 | -1.25 | -1.49 | -1.10 |
| *HIF1A* | -1.56 | 1.09 | -1.10 | -1.16 | 1.06 | 1.11 | 1.23 |
| *STAT3* | -1.32 | -1.04 | -1.66 | -1.26 | 1.25 | -1.05 | 1.58 |
| *UCP2* | -1.29 | -1.11 | 1.17 | -1.50 | -1.68 | 1.05 | -1.12 |
| *HIGD2A^≠^* | -1.27 | -1.06 | 1.61 | 1.17 | -2.36 | -1.72 | **-2.77** |
| *WASH1* | -1.27 | -1.20 | -1.42 | 1.20 | 1.06 | -1.60 | -1.13 |
| *BIN3* | -1.16 | -1.09 | -1.01 | -1.29 | -1.28 | -1.00 | 1.01 |
| *PRKAR1A* | -1.14 | -1.03 | -1.10 | -1.24 | -1.05 | 1.07 | 1.18 |
| *NDUFB11* | -1.13 | -1.09 | -1.19 | -1.11 | 1.02 | -1.06 | 1.13 |
| *ANXA5* | -1.05 | -1.02 | 1.04 | -1.06 | -1.09 | 1.01 | -1.03 |
| *SMS^≠^* | 1.08 | 1.02 | -1.05 | -1.19 | 1.14 | 1.11 | **1.27** |
| *PPP2R5E* | 1.13 | 1.08 | 1.12 | 1.02 | 1.01 | 1.11 | -1.01 |
| *NAP1L1* | 1.14 | 1.16 | 1.06 | 1.09 | 1.11 | 1.08 | 1.02 |
| *PTEN* | 1.15 | -1.02 | 1.03 | 1.14 | 1.13 | 1.02 | -1.01 |
| *LOC646214* | 1.16 | 1.00 | 1.01 | 1.20 | 1.20 | 1.17 | 1.08 |
| *LMCD1* | 1.21 | 1.19 | 1.04 | 1.17 | 1.17 | 1.32 | 1.13 |
| *CAMSAP2* | 1.23 | 1.09 | 1.26 | 1.21 | 1.21 | -1.02 | -1.12 |
| *TSC2* | 1.26 | -1.37 | 1.15 | -1.28 | -1.35 | 1.09 | -1.05 |
| *FLJ45139* | 1.33 | 1.36 | 1.40 | 1.29 | -1.05 | 1.03 | -1.36 |
| *CHSY3* | 1.72 | 1.17 | 1.33 | 1.67 | 1.27 | 1.01 | -1.32 |
| *OVOS* | 1.72 | -1.10 | 1.52 | 1.35 | 1.12 | 1.27 | -1.20 |
|  |  |  |  |  |  |  |  |

^≠^ potential role of SFA/MUFA ratio in the regulation of gene expression.

Abbreviations: **c9**. t11CLA - cis-9.trans-11-CLA; **t10**. c12CLA- trans-10.cis-12-CLA. **S/M**. changed SFA/MUFA ratio.
